# Deep learning-based algorithm for postoperative glioblastoma MRI segmentation: a promising new tool for tumor burden assessment

**DOI:** 10.1186/s40708-023-00207-6

**Published:** 2023-10-06

**Authors:** Andrea Bianconi, Luca Francesco Rossi, Marta Bonada, Pietro Zeppa, Elsa Nico, Raffaele De Marco, Paola Lacroce, Fabio Cofano, Francesco Bruno, Giovanni Morana, Antonio Melcarne, Roberta Ruda, Luca Mainardi, Pietro Fiaschi, Diego Garbossa, Lia Morra

**Affiliations:** 1 Neurosurgery, Department of Neuroscience, University of Turin, via Cherasco 15, 10126 Turin, Italy; 2 Dipartimento di Automatica e Informatica, Politecnico di Torino, Turin, Italy; 3 Department of Neurosurgery, Barrow Neurological Institute, St. Joseph’s Hospital and Medical Center, Phoenix, AZ USA; 4 Neurosurgery, University of Messina, Messina, Italy; 5 Neurooncology, Department of Neuroscience, University of Turin, Turin, Italy; 6 Neuroradiology, Department of Neuroscience, University of Turin, Turin, Italy; 7 Dipartimento di Elettronica, Informazione e Bioingegneria, Politecnico di Milano, Milan, Italy; 8 IRCCS Ospedale Policlinico S. Martino, Genoa, Italy; 9 Dipartimento di Neuroscienze, Riabilitazione, Oftalmologia, Genetica e Scienze Materno-Infantili, Univeristy of Genoa, Genoa, Italy

**Keywords:** Glioma, Glioblastoma, Magnetic resonance imaging, Deep learning, Machine learning, Segmentation

## Abstract

**Objective:**

Clinical and surgical decisions for glioblastoma patients depend on a tumor imaging-based evaluation. Artificial Intelligence (AI) can be applied to magnetic resonance imaging (MRI) assessment to support clinical practice, surgery planning and prognostic predictions. In a real-world context, the current obstacles for AI are low-quality imaging and postoperative reliability. The aim of this study is to train an automatic algorithm for glioblastoma segmentation on a clinical MRI dataset and to obtain reliable results both pre- and post-operatively.

**Methods:**

The dataset used for this study comprises 237 (71 preoperative and 166 postoperative) MRIs from 71 patients affected by a histologically confirmed Grade IV Glioma. The implemented U-Net architecture was trained by transfer learning to perform the segmentation task on postoperative MRIs. The training was carried out first on BraTS2021 dataset for preoperative segmentation. Performance is evaluated using DICE score (DS) and Hausdorff 95% (H95).

**Results:**

In preoperative scenario, overall DS is 91.09 (± 0.60) and H95 is 8.35 (± 1.12), considering tumor core, enhancing tumor and whole tumor (ET and edema). In postoperative context, overall DS is 72.31 (± 2.88) and H95 is 23.43 (± 7.24), considering resection cavity (RC), gross tumor volume (GTV) and whole tumor (WT). Remarkably, the RC segmentation obtained a mean DS of 63.52 (± 8.90) in postoperative MRIs.

**Conclusions:**

The performances achieved by the algorithm are consistent with previous literature for both pre-operative and post-operative glioblastoma’s MRI evaluation. Through the proposed algorithm, it is possible to reduce the impact of low-quality images and missing sequences.

## Introduction

Glioblastoma is the most common adult malignant primary brain tumor [1, 2]. Despite the advances in therapeutic options and management, survival of patients with glioblastoma remains around 15–18 months, with a 5-year overall survival of approximately 5% [3–5]. In order to improve the prognosis of the disease, research attempts are focused on new tools to individualize the therapeutic approach according to clinical, radiological, and molecular characteristics. Magnetic resonance imaging (MRI) is an essential tool for glioblastoma evaluation, providing necessary information to set up the best therapeutic strategy, both in preoperative decisions and in postoperative management, for each individual patient. Furthermore, MRI-based segmentation allows for volumetric assessment of different tumor components and, thus, precise surgical planning [6, 7]. The current standard of MRI segmentation for tumoral lesions relies on manual measurements; however, this method is not only time-consuming, but also not reproducible due to inter-operator variability [7–13]. For this reason, semi-automatic and completely automatic segmentation algorithms have been developed and evaluated in previous studies [14, 15]. With the recent development of Deep Convolutional Neural Networks (DCNNs) such as U-Net, now widely used for segmentation of medical images, the level of accuracy has increased [16].

Growing efforts to apply Artificial Intelligence (AI) to medical imaging analysis has resulted in the availability of larger datasets and improvements to software performances [17]. For research purposes, MRIs have been collected and benchmarked in large datasets, such as the Brain Tumor Image Segmentation (BraTS) dataset [9, 14].

Despite the good results obtained in the evaluation of preoperative images [7, 18–20], AI MRI segmentation is limited in its postoperative evaluation and external validity [7, 10]. Limitations in postoperative MRI evaluation are partly due to artifacts, caused by blood and air in the resection cavity, and logistical issues in collecting data regularly from the same patient during follow-up. Moreover, the results obtained from most of the algorithms are not easily reproducible in the clinical context since they are frequently trained on curated and standardized datasets that do not include suboptimal quality images.

Obtaining a reliable system for postoperative automatic segmentation of MRI would bring many benefits to clinical practice. First, automatic segmentation would guarantee a fast and objective evaluation of MRI. This would be useful in optimizing and personalizing administration of treatment specific to unique features present in each case. Additionally, a well-trained algorithm may be able to perceive more than human experts can, possibly obtaining further information about the likely disease progression. Finally, it is important to train the algorithm to be considerate of common clinical practice issues, such as the heterogeneity of data and the frequent lack of MRI sequences (non-contrast enhanced T1 and T2 are not always included in MRI datasets). Hence, the main objective of the study is to obtain a valid segmentation algorithm in the postoperative scenario that can be a useful tool in the assessment of tumor volumes during the oncologic follow up of glioblastoma patients, moving AI closer to clinical practice maintaining the level of reliability previously obtained in other studies.

## Methods

### Dataset

The neurosurgery unit of the hospital Molinette (AOU Città della Salute e della Scienza di Torino) acquired a dataset comprising 71 patients who underwent surgery at the institute, with histologically confirmed Glioblastoma Grade IV. All MRI scans acquired in situ were accessible by default on the hospital SYNAPSE® Mobility PACS system. Scans acquired in a different center were routinely uploaded onto the BRAINLAB© neuronavigation system of the neurosurgery department. At the time of hospitalization, written consent for personal, biological, and radiological data processing for scientific purposes was explicitly asked and registered on the InterSystem TrakCare©information system. The present study was approved by the local Institutional Review Board (n. 00162/2022). The following exclusion criteria were applied: underage subject (< 18 years old), absence of T1 contrast enhancing (T1ce) or fluid attenuated inversion recovery (FLAIR) MRI sequences, postoperative complications (e.g., hemorrhage or abscess) which could possibly invalidate the segmentation, or absence of histological confirmation. For each patient, one or more scans were available, including preoperative and postoperative images, with acquisition-time ranging from immediately after the surgery (max 48 h) to 12 months later. For each MRI scan, a volumetric T1ce sequence and a FLAIR sequence were added to the radiological database.

The data were anonymized before processing, as instructed by the EU General Data Protection Regulation, using the specific function available in the HOROS©DICOM image viewer.

### Semi-automatic segmentation

Both preoperative and postoperative segmentations were performed semi-automatically through the SmartBrush feature of the Cranial Planning workflow inside the BRAINLAB© neuronavigation system (Build 3.3.1.404). The volumetric representation was reconstructed by the software by combining the semi-automatic segmentations in the axial, coronal, and sagittal planes. The axial view was then extracted and, if necessary, manually adjusted. The process was carried out individually by 4 neurosurgeons, and 1 medical student. All the segmentations were revised and confirmed by a senior neurosurgeon with 25-year expertise in neurooncology and a neuroradiologist.

The segmented classes consisted of tumor core (TC), enhancing tumor (ET), and whole tumor (WT, including ET and FLAIR hyperintensity) for the preoperative images; resection cavity (RC), gross tumor volume (GTV, including RC and ET) and WT (including RC, ET and FLAIR hyperintensity) were the classes for the postoperative images. For both preoperative and postoperative cases, whole tumor segmentation was performed on the FLAIR sequence, whereas the remaining two classes on the T1ce one.

### Harmonization

Since the model (cfr. next section for architectural specifications) is pre-trained on the BraTS 2021 dataset, in order to allow for processing postoperative MRIs collected from the Molinette Hospital, it is necessary to harmonize them in a BraTS-like manner by implementing both uniform atlas registration and skull-stripping.

The whole pipeline is available through the Cancer Imaging Phenomics Toolkit [22, 23], but it was only followed for the SRI-24 atlas co-registration portion.

Therefore, co-registration to the SRI-24 template with uniform isotropic resolution (1 mm^3^) was performed [21]. Skull-stripping was instead performed through SynthStrip [24].

**Fig. 1:**
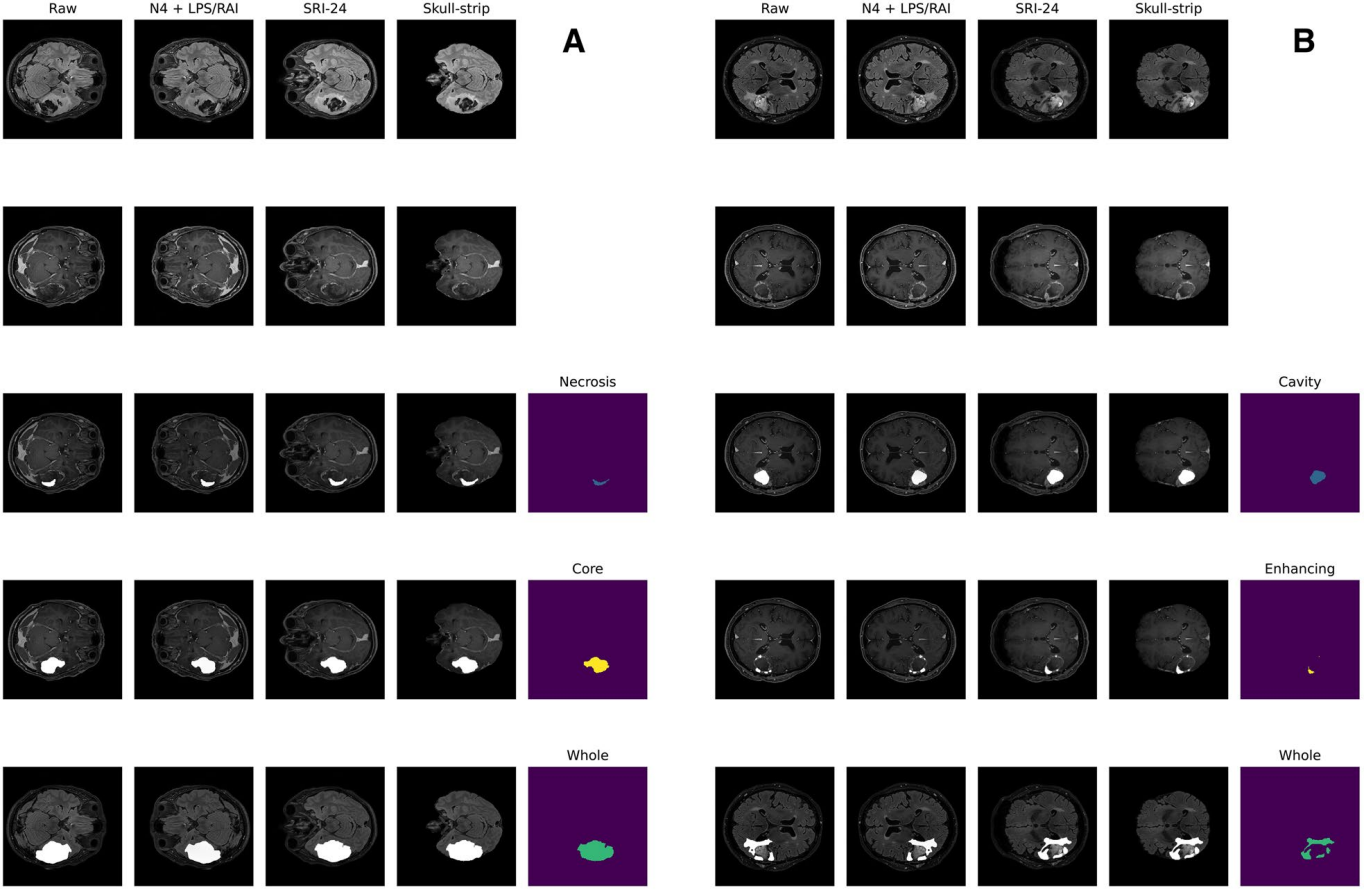
Application of the final BraTS harmonization pipeline for the patients in the Molinette dataset, comprising SRI-24 co-registration performed with CaPTk and skull-stripping by SynthStrip. **A** Preoperative (first and whole row—FLAIR; second, necrosis, core rows—T1ce). **B** Postoperative (first and whole rows—FLAIR; second, cavity, enhancing rows—T1ce). The previous figure comprises in detail all the steps required in the pipeline: N4 bias correction for magnetic field inhomogeneities, LPS/RAI voxel re-orientation, SRI-24 co-registration and skull-stripping. The first two rows show the harmonization pipeline for two examples coming from the BraTS dataset (left, preoperative) and the Molinette dataset (right, postoperative). It is worth noticing that the Atlas co-registration modifies the depth dimension (i.e., the number of slices), therefore the most similar interpolated slice is here shown for visual representation

The CaPTk pipeline required the presence of four scan modalities—FLAIR, T1, T1ce, and T2—but only FLAIR and T1ce were always present in the dataset. Hence, the burned-in segmentations, i.e., MRIs with class segmentation “burned-in” inside of them as high-intensity regions, were used as T1 (burned-in T1ce) and T2 (burned-in FLAIR). SynthStrip was then applied to extract the brain mask from the original end-of-pipeline T1ce scan, with the resulting mask adopted for all remaining scans (Fig. 1).

Once both raw and burned-in data were registered to the SRI-24 atlas and skull-stripped, all classes were extracted by retrieving high-intensity regions. The complete segmentation was built by overlapping such extracted regions, i.e., by joining the necrosis/RC (respectively, for pre/post-operative scans), the ET, and the peripheral edema.

### Proposed architecture

The selected architecture was the winning contribution to the BraTS 2021 challenge, a 3D U-Net derived from the nnU-Net framework [25]. Therefore, following the official NVIDIA GitHub repository, the nnU-Net foundational skeleton, the preprocessing and the training schedule were implemented. Pre-training on preoperative BraTS dataset was performed for 150 epochs while all other relevant hyperparameters such as the ones governing data augmentation, regularization, cross-validation or post-processing, were kept as presented in the work by Furtega et al. [25] By processing the complete segmentations described in the last section into the three overlapping classes previously presented, ground truth for the training process was obtained. The network was trained on a high performance computing (HPC) server using one 32G V100 nVIDIA GPUs. One of the challenges do be the tackled was the varying number of sequences available in clinical studies. Indeed, T1 and T2 sequences were frequently absent in the dataset, whereas existing architectures for preoperative sequences are trained to produce the segmentation based on four sequences (which are T1ce, FLAIR, T1, T2). This issue can be tackled in several ways: on one hand, one could simply remove sequences that are not commonly available in all studies to train the postoperative segmentation network.

**Fig. 2:**
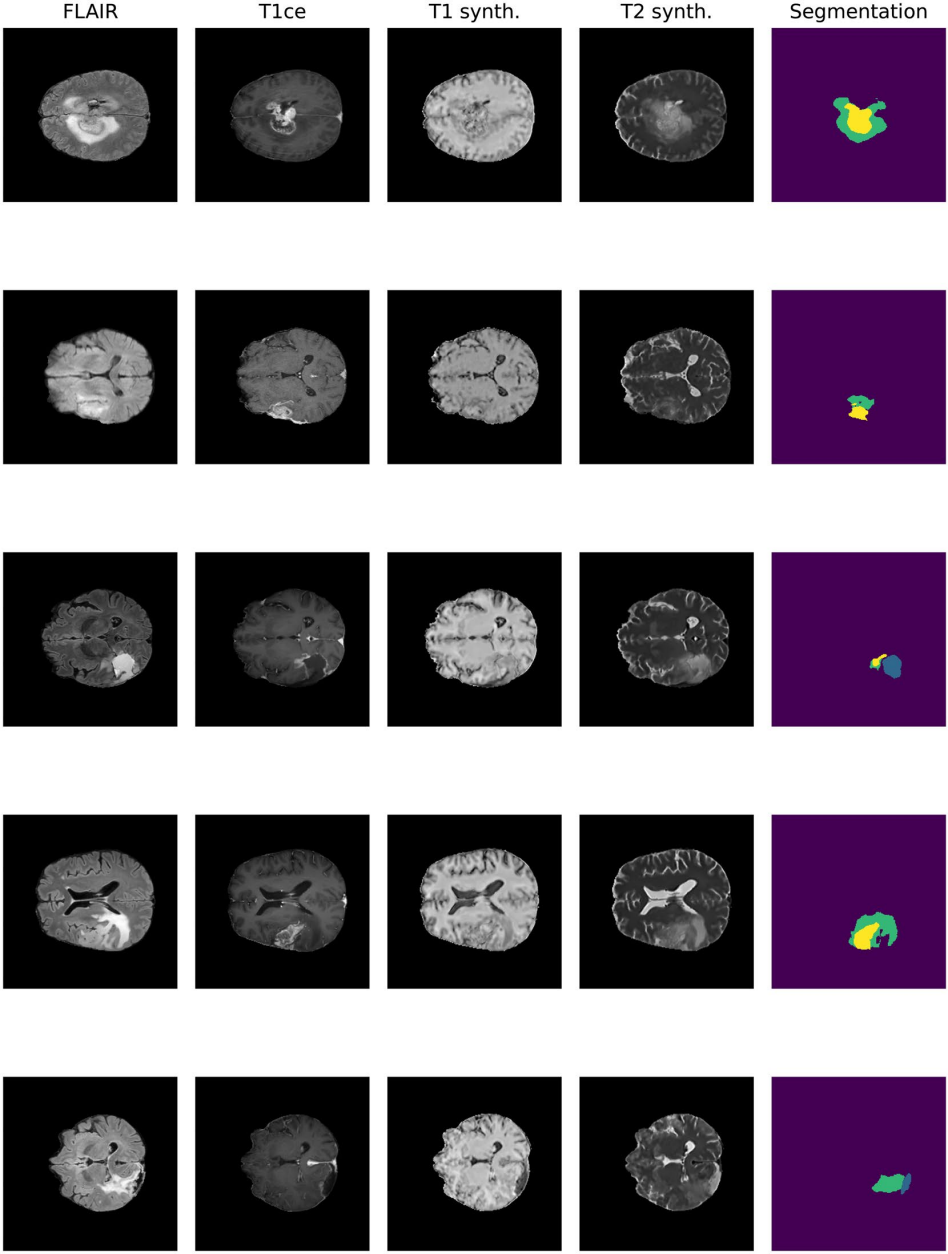
Application of the IMT U-Net architecture to postoperative MRI from the Molinette Hospital dataset. The different rows refer to representative slices from five randomly extracted patients (blue: cavity, yellow: enhancing, green: whole)

On the other hand, one could seek to artificially synthesize the missing sequences using image modality translation (IMT) techniques (Fig. 2).

In this work, this last proposal was chosen: a 2D U-Net, receiving as input single MRI slices, was built in order to learn from the BraTS dataset the non-linear mapping between available and desired sequences.

These artificial sequences, although in a way “hallucinated” by the network, could be used in place of missing sequences, thus allowing the network to be trained on studies with a variable number of sequences. In this study, both strategies were compared. The modified U-Net structure originally proposed by Osman and Tamam [26] was implemented to artificially synthesize the missing sequences, stacking T1ce and FLAIR, hence exploiting information from both of them, instead of simply using a single sequence as input. The network was trained for 50 epochs with mixed precision on the BraTS 2021 dataset. The underlying U-Net required 2D inputs, therefore for each iteration a 3D scan was selected and a random selection of 64 2D slices (being this a reasonable amount of slices to balance computational cost and learning performances), possibly flipped along the two dimensions, was extracted and fed as a batch. The network was trained with the default Adam optimizer with a learning rate of 0.0003, which decays following a cosine schedule. Output images were shaped 224 × 224 so post-processing was applied to resize, re-orient and pad them in their BraTS form. Gaussian sharpening was also applied as further data augmentation. The synthesis was carried out using the T1ce MRI acquisition, as the additional use of non-volumetric FLAIR scans would eventually generate noisy outputs.

The network proposed by Futrega et al. [25] and pre-trained on preoperative brain tumor segmentation, was fine-tuned on the Molinette postoperative dataset to reap the benefit of Transfer Learning (TL) [27] . Several TL—strategies have been investigated to experimentally determine the best balance between underfitting and overfitting. All configurations with frozen layers in depth levels 1–7 have been studied, but no performance improvement or relevant advancement were observed. As a result, fine-tuning, consisting of tuning all weights from the pre-trained model, was chosen for the final experiments.

Fine-tuning on postoperative cases was performed for 200 epochs with a learning rate of 0.0007, which decays following a cosine schedule having 0.25 as cosine cycles parameter. The other relevant hyperparameters were kept the same as in the preoperative cases. A more aggressive data augmentation strategy was adopted by increasing the probability of applying a given transformation from 0.15 to 0.5: as suggested by Zhang et al., a more aggressive data augmentation might help in improving the model´s generalizability when trained on a small dataset [29].

### Performance analysis

#### Cross-validation

A fivefold cross-validation was performed on the Molinette dataset with the purpose of maximizing the use of available data, while providing a more reliable estimation [27]. The dataset was split at the patient level to ensure that different folds are statistical independent, i.e., the network is not trained and tested on scans from the same patient. Thus, it was possible to estimate how well the model generalizes on never-before-seen individuals.

Non-volumetric scans were included in order to have a dataset representative of common clinical scenarios. Nevertheless, patients with non-volumetric scans were only included in the training set and not in the validation set, since nowadays non-volumetric scans are less frequent in clinical practice, and thus it was better to test only on standard scans without compromising the validation of the algorithm.

**Fig. 3:**
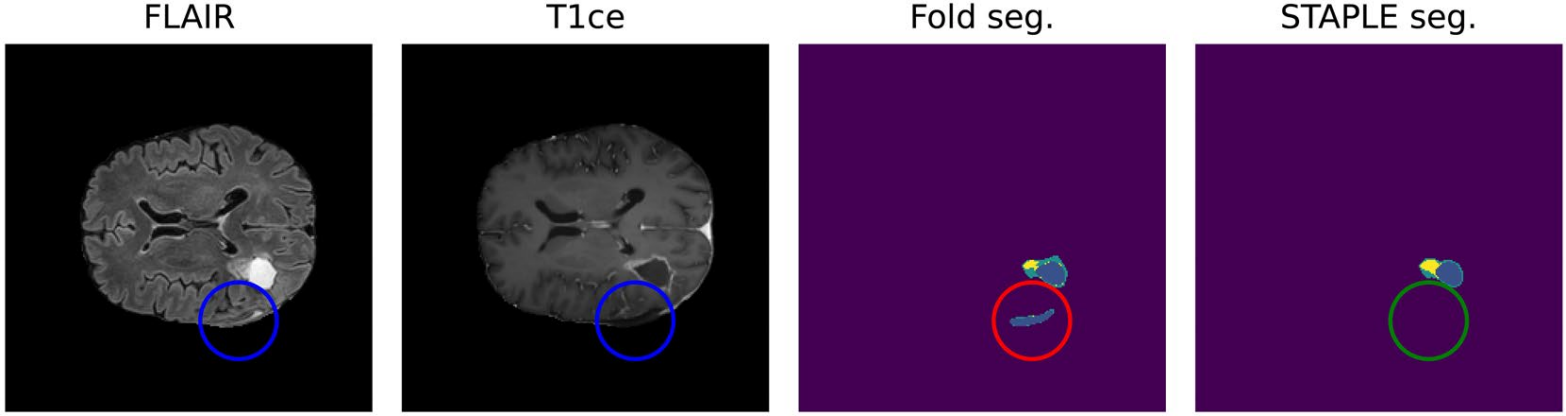
Positive effect of STAPLE fusion for resection cavity segmentation. Results obtained from the fivefold cross-validation process (fold seg.) are merged by the STAPLE algorithm to obtain a final result (STAPLE seg.). The figure shows, as an example, how the STAPLE convergence is able to recognize oversegmentation of a hypointense region misclassified as resection cavity (blue: cavity, yellow: enhancing, green: whole)

Finally, STAPLE algorithm was used to evaluate and compare all output segmentations returned by the fivefold cross-validation [28] (Fig. 3).

#### Post-processing

Post-processing was applied in order to bring back the three overlapping regions to the default ones of edema, ET, and RC. Specifically, if the probability of a voxel being classified as either GTV or edema was ≤ 0.45, it was classified as background. Else, if the probability of being classified as GTV was ≤ 0.4, then the voxel was classified as edema. If the probability of being a GTV voxel was > 0.4 and the same held for the probability of being classified as RC, then the voxel was identified as such (or simply ET if the latter was not true). Moreover, any connected component identified as RC smaller than 16 voxels with an overall probability smaller than 0.9 was ignored and classified as ET instead (Fig. 3). These values were determined via a grid search on the 5 folds, starting from the parameters proposed by Futrega et al. [25] for the preoperative case.

## Results

The dataset comprised 237 MRIs (71 preoperative and 166 postoperative) from 71 patients. All sequences were acquired on 1.5 or 3.0 Tesla, with 230*230 mm2 or less FOV and matrix size 512 × 512 or 1024 × 1024. The characteristics and number of those MRIs are summed up in Table 1.

**Table 1:** Quantitative description of the postoperative dataset from Molinette Hospital (NC: no cavity, NE: no enhancing, C: complete, NV: non-volumetric)

Number of MRI scans	Number of patients	MRI scan type	Total
1	22	NC: 5 | NE: 7 | C: 10	22 (NV: 4)
2	25	NC: 3 | NE: 14 | C: 33	50 (NV: 9)
3	9	NC: 8 | NE: 8 | C: 11	27 (NV: 12)
4 +	15	NC: 12 | NE: 22 | C: 33	67 (NV: 21)
Total	71	NC: 28 | NE: 51 | C: 87	166 (NV: 46)

### Preoperative results

Evaluation was performed on two different configurations of available scans: the “complete” one, i.e., the one comprising all four sequences (FLAIR, T1, T1ce and T2), and the “most-informative subset” one, i.e., the one comprising just FLAIR and T1ce sequences. The complete configuration included as T1 and T2 the ones artificially synthesized through the 2D U-Net IMT method described above.

DS values and H95 are obtained by comparing results obtained from the automatic segmentation performed by the trained algorithm with those obtained by human experts using Brainlab software.

**Fig. 4:**
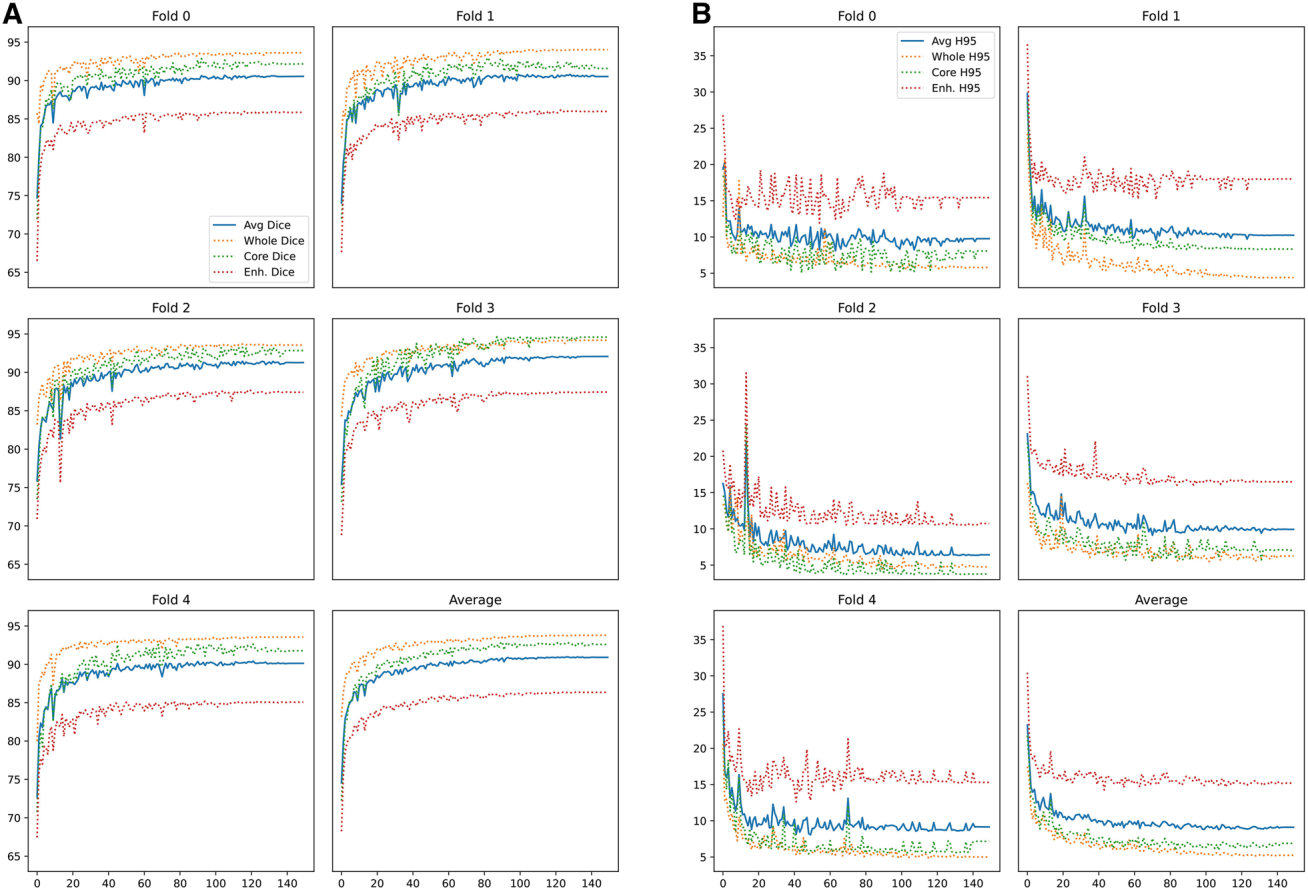
Illustrates the trend DICE scores (**A**) and Hausdorff 95 distance scores (**B**) on the “complete” configuration for preoperative segmentation. y axis: DS and H95 values; x axis: each point represents a patient from the Molinette hospital dataset. Segments considered are TC, ET and WT

The overall mean DS and H95 are 91.09 (± 0.60) and 8.35 (± 1.12), respectively, for the “complete” subset and 90.77 ± 0.67 and 8.35 ± 1.12 for the “most-informative” one (Fig. 4).

Results show that, even if optimal performance is obtained on the “complete” configuration (overall DS: 91.09 ± 0.60), reliable outcomes are also achieved with the two most informative MRI scans (overall DS: 90.77 ± 0.67).

Results obtained during a fivefold cross-validation of the BraTS 2021 dataset are in line with those presented by Futrega et al. (Dice Score (DS): 91.63). The result obtained (DS: 91.09) confirms the foundation of such an nnU-Net implementation for preoperative brain tumor segmentation in MRI.

### Postoperative results

**Fig. 5:**
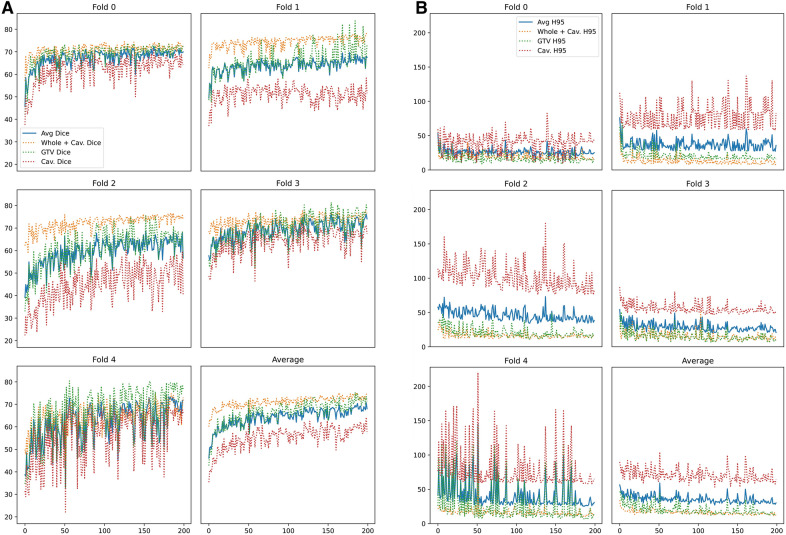
Illustrates the trend DICE scores (**A**) and Hausdorff95 distance scores (**B**) on the “complete” configuration for postoperative segmentation. The graphs show trend DICE scores for postoperative segmentation, considering the RC (Cav. Dice and Cav. H95), the GTV (GTV Dice and GTV H95), the whole tumor (WT: Whole + Cav. Dice and Whole + Cav. H95), the average result (avg). The fivefold are considered separately and altogether

As for preoperative scans, evaluation was performed for “complete” subset, and the “most-informative subset” one. The overall mean DICE score was 72.44 (± 3.49), while Hausdorff 95 distance was 23.43 (± 7.24) for the postoperative “most informative subset” assessment, as shown in Fig. 5. Similar results are obtained with the “complete” subset (DS: 72.31 ± 2.88, HD95: 23.85 ± 7.20) (Fig. 5).

Remarkably, the RC segmentation obtained a mean DS of 63.52 (± 8.90) in postoperative MRIs (Table 2).

**Table 2:** Experimental dice scores and Hausdorff 95 distances obtained during fivefold cross-validation on the postoperative Molinette Hospital dataset for the two available modalities configurations

Model	Available modalities	All (Dice score)	GTV (Dice score)	Cavity (Dice score)	Mean (Dice score)	All (Hausdorff 95 distance)	GTV (Hausdorff 95 distance)	Cavity (Hausdorff 95 distance)	Mean (Hausdorff 95 distance)
*Fold 0*	FLAIR, T1ce	74.45	74.52	69.77	72.91	15.20	9.96	8.78	11.31
	FLAIR, T1, T1ce, T2	73.07	72.70	71.23	72.33	14.77	10.70	12.07	12.51
*Fold 1*	FLAIR, T1ce	77.80	74.89	54.78	69.16	8.09	16.36	59.18	27.88
	FLAIR, T1, T1ce, T2	76.62	82.39	50.05	69.69	7.99	15.87	57.70	27.19
*Fold 2*	FLAIR, T1ce	76.10	71.41	55.93	67.81	11.40	11.60	75.23	32.74
	FLAIR, T1, T1ce, T2	75.07	75.48	55.85	68.80	13.71	12.55	75.98	34.08
*Fold 3*	FLAIR, T1ce	77.16	81.23	71.82	76.74	7.83	7.03	47.00	20.62
	FLAIR, T1, T1ce, T2	76.86	81.38	71.96	76.73	7.83	6.59	46.30	20.24
*Fold 4*	FLAIR, T1ce	77.02	80.43	69.31	75.59	9.34	6.56	57.94	24.61
	FLAIR, T1, T1ce, T2	73.93	79.58	68.50	74.00	10.00	6.96	58.70	25.22
*Mean* + *-STD*	FLAIR, T1ce	76.51 ± 1.16	76.50 ± 3.75	64.32 ± 7.38	72.44 ± 3.49	10.37 ± 2.72	10.30 ± 3.56	49.63 ± 22.32	23.43 ± 7.24
	FLAIR, T1, T1ce, T2	75.11 ± 1.48	78.31 ± 3.67	63.52 ± 8.90	72.31 ± 2.88	10.86 ± 2.88	10.53 ± 3.49	50.15 ± 21.27	23.85 ± 7.20

Again, the lack of T1 and T2 has not been proven to significantly impact the results achieved. The addition of synthetic T1 and T2 scans did not improve the overall segmentation, as suggested by previous literature.

**Fig. 6:**
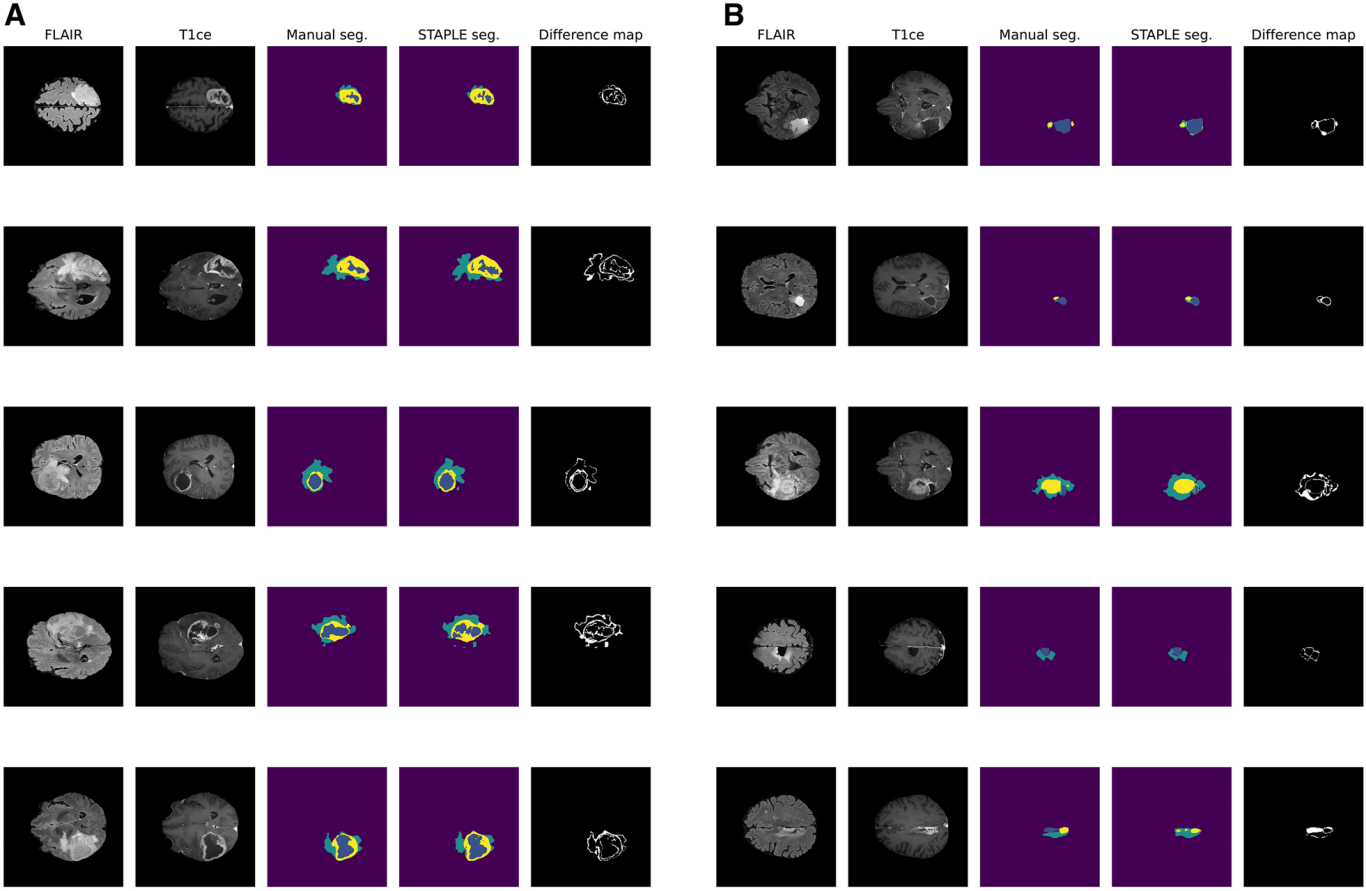
Examples of preoperative (**A**) and postoperative (**B**) segmentation on 4 patients randomly extracted from the Molinette dataset

Graphical examples of automatic segmentation with difference map from manual segmentation, both for preoperative and postoperative MRI from Molinette database, are shown, respectively, in Fig. 6A and B.

## Discussion

As previously stated, currently the gold standard for volumetric assessment is manual segmentation. Numerous efforts have been taken to improve volumetric assessment and segmentation of the tumoral lesion in the preoperative context [18–20, 30].

Available automatic algorithms were developed mainly for preoperative images; this results in low reliability for postoperative assessment [6].

The practical reason behind this is the intrinsic difficulty in postoperative MRI segmentation [7, 30]. In fact, the RC is frequently a source of artifacts in the MRI because of blood residuals and air bubbles [31, 32]. In addition to this, brain anatomy may be partly altered as a consequence to the surgical act, the post-surgical edema and the tumor itself [32]. These problems lower the accuracy of available algorithms in obtaining postoperative evaluation of MRI, in addition to logistical issues concerning regular post-surgical follow-up [33]. Nevertheless, some studies recently reported good accuracy in postoperative segmentation of MRI, though it is still far from the level of accuracy achieved in preoperative evaluation [7, 33].

Another limit of the available algorithms is that they are often trained on cured and standardized datasets that do not include low-quality images. Though this selection bias makes the training process easier, it is not as easily transferable to real-world clinical practice. In fact, suboptimal quality of data is very common in clinical practice, including non-volumetric scans, missing sequences, and artifacts [34].

In this study, we aimed to train an AI algorithm for the postoperative MRI evaluation of glioblastoma in order to prospectively introduce this tool in clinical practice as support for the decision-making process. For this reason, the MRI database used for the training is representative of the real-world clinical scenario, frequently including heterogeneous and incomplete data. Even if a few cases were excluded from this study (e.g., in case of postoperative abscess or hemorrhage), we did not apply restrictive inclusion criteria concerning the quality of the available data in order not to affect the results with selection bias. Having images with different resolution (since FOV may differ) and contrast (1.5 T or 3 T) makes the learning process more complex but results in increased adaptability of the algorithm to different clinical scenarios. In fact, MRI acquisition protocols are slightly different according to the institution and may change over time within the same hospital.

Low-quality images were also included (e.g., non-volumetric imaging). We did not benefit from incorporating non-volumetric imaging in the training phase, since its inclusion in the dataset creates a difficult scenario for the algorithm to be correctly classified. The benefits resulting from the incorporation of these data are related to clinical applicability of the algorithm, since the presence of non-volumetric images is related to old acquisition protocols, but their presence in the clinical scenario was relevant, accounting for almost 25% of the MRIs collected in the Molinette database. As a consequence, this data was not excluded from the study as it would limit prospective application of the algorithm in clinical practice.

Moreover, postoperative images have different acquisition times given the time-course of the disease and the treatment schedule. This means that the postoperative MRI database contains images from different points in time: immediate postoperative, before and after adjuvant treatment, and regular follow-up. Herein, the algorithm is exposed to different biological entities such as post-surgical residual, RC, progressively growing lesion, and edema.

Herein, the results achieved are similar to the ones reported in other studies, considering both preoperative (average DS: 91.09 ± 0.60) and postoperative (average DS: 72.31 ± 2.88) evaluation. In particular, the DS is comparable if not slightly better than the work by Gazit et al. [35] (average DS 0.71) and by Nalepa et al. (average DS 0.69) [36] and lower than Chang et al. (average DS 0.76), a multicenter study with a very large number of patients, although no results are reported for resection cavity which was the volume with the most difficulty in segmentation [33].

From the results obtained, it is evident that the accuracy in the postoperative setting is still far away from that in the preoperative scenario. This contrast in accuracy is especially remarkable for the RC segmentation, with a mean DS of 63.52 ± 8.90. This element causes both cases of hyper-segmentation, including adjacent regions, and sub-segmentation, excluding some parts of the cavity. Nevertheless, the evaluation of the RC is complex with less accurate results even for expert human operators performing manual segmentation. Possible reasons behind poor segmentation of cavity may be the presence of air or blood products in the resection cavity. They are only present in the first postoperative MRI (as a consequence of recent surgical procedure) and therefore the numbers were too small for adequate algorithm training. A larger cohort and better characterization of these confounding effects, particularly through a temporal stratification, may allow effective stratification of blood products and air sacs and lead to a better ability to manage these cases.

The novelty of the proposed method is to use strategies, such as TL or STAPLE segmentation, to overcome low sample numbers and heterogeneous or non-volumetric MRI images, making the algorithm closer to clinical practice. Particularly the use of TL, which coenables the algorithm to learn from the preoperative images, where there are very large and more standardized databases, to use the information acquired in postoperative segmentation.

Furthermore, the level of accuracy reached in this study was moderately improved by the application of data augmentation, cross-validation and an ensemble of models aggregated through the STAPLE algorithm in order to compensate for the limited amount of data. Another challenge for applying automatic segmentation in clinical practice is the variable number of sequences available. IMT is a technique that takes information from existing sequences to create the missing ones, but it is still at an experimental level. In this study, IMT architecture from Osman et al. [26] was applied to T1ce sequences to create T1 and T2 whenever they were not available in the Molinette database. In preoperative segmentation, the additional presence of these sequences proved to be nonessential but they slightly improved (non-significatively) the performance of the algorithm. In our study, we did not observe any benefits associated with IMT, unlike suggested by previous literature [37], resulting in the least effective strategy of those used. However, it is possible that with larger or more diverse datasets the quality of the synthesized images could be improved, especially in the postoperative setting.

In addition, the impact in terms of time sparing that the use of this algorithm might entail in clinical practice should not be overlooked. Segmenting manually or even semi-automatically is a time-consuming process in itself, and this problem is exacerbated in the postoperative setting where there are many follow-up MRIs to segment, with the previously reported difficulties due to the presence of artifacts post-surgery or following adjuvant treatments.

### Limits of the study

Institutional studies with private datasets are essential to scientific and informatic research, but they have some limitations [38]. Literature reports that models developed and tested with data from one collection hardly achieve similar results when applied to data from a different institute [39]. It is therefore advisable to corroborate the results from this study with multi-institutional data, consequently increasing the level of reliability.

Even if selection bias wanted to be limited, some cases were excluded from the postoperative MRI database, e.g., hemorrhage and abscess cases.

In addition, several studies highlight that reference standards based on the expertise of radiologists are not completely objective [40]. It is reported that the number of operators performing the segmentation should be at least three [38], while, in this study, the manual segmentation was performed by four neurosurgeons, one medical student and revised by a senior neurosurgeon and a neuroradiologist in order to overcome interobserver variability.

A further limitation in the proposed work is the final post-processing pipeline proposed to bring back labels to tumoral segments of the postoperative evaluation (edema, enhancing tumor and resection cavity). Even if the parameters are obtained by averaging grid-search outputs, the limited amount of data decreases the reliability of these values.

As the training phase influences the outcomes of the algorithm, quality assessment of MRIs used in this step would be helpful. Moreover, results would be more accurate if the FLAIR sequence was always volumetric, however, the purpose of this work was to avoid selection bias of data to get an algorithm reliable in clinical practice. For this reason, the possible improvement relies on more accurate protocols of MRI acquisition in common clinical practice and not on image selection for research studies.

### Future perspectives

Due to the benefits granted by informatic tools and strategies, our results are in line with the existing literature on this topic. Different from previous studies, this work is not biased by restrictive inclusion/exclusion criteria for MRI scans. Therefore, we present this work as a starting point to apply AI to clinical practice for glioblastoma with remarkable reliability both in the preoperative and postoperative context.

Future studies should involve multiple institutions, allowing for an increase in the sample size of the database overall and of glioblastoma postoperative MRIs acquired from different protocols and machines. Moreover, experimental techniques such as IMT could be refined, adding greater support to the algorithm. The elimination of non-volumetric scans and low-quality imaging from clinical practice would be essential not only for research purposes but also for future clinical application of the AI technologies. All of these initiatives may improve the AI algorithm performance and lead to clinically reliable use of AI in glioblastoma evaluation.

Finally, working with AI requires simultaneous specialized technical competences and a comprehensive view of the clinical scenario. Thus, it is advisable to face the current limitations of biological, clinical, logistical, and technical issues within the analysis from a multidisciplinary point of view. This outlook highlights the importance of clear communication between the neurosurgical team and the engineers in searching for appropriate solutions.

## Conclusions

This study sought to create a reliable tool for automatic postoperative MRI segmentation of glioblastoma, making it closer to a realistic clinical setting. The algorithm obtained still has some limitations, but the results of the study are in line with the existing literature. Moreover, the authors chose to train the algorithm to be reliable in clinical practice, especially in cases of missing sequences or low-quality images. Some strategies have been proposed in this work to overcome these limitations, with promising results. In the future, the remaining challenges ahead may be faced by increasing the dataset size and implementing innovative technical strategies.

## Data Availability

The data that support the findings of this study are not openly available due to reasons of sensitivity and are available from the corresponding author upon reasonable request.
